# Patterns of *Saccharina latissima* Recruitment

**DOI:** 10.1371/journal.pone.0081092

**Published:** 2013-12-12

**Authors:** Guri Sogn Andersen

**Affiliations:** Department of Biosciences, University of Oslo, Oslo, Norway; University of New South Wales, Australia

## Abstract

The lack of recovery in Norwegian populations of the kelp *Saccharina latissima* (Linnaeus) C. E. Lane, C. Mayes, Druehl & G. W. Saunders after a large-scale disturbance that occurred sometime between the late 1990s and early 2000s has raised considerable concerns. Kelp forests are areas of high production that serve as habitats for numerous species, and their continued absence may represent the loss of an entire ecosystem. Some *S. latissima* populations remain as scattered patches within the affected areas, but today, most of the areas are completely devoid of kelp. The question is if natural recolonization by kelp and the reestablishment of the associated ecosystem is possible. Previous studies indicate that a high degree of reproductive synchrony in macrophytes has a positive effect on their potential for dispersal and on the connectivity between populations, but little is known about the patterns of recruitment in Norwegian *S. latissima*. More is, however, known about the development of fertile tissue (sori) on adult individuals, which is easily observed. The present study investigated the degree of coupling between the appearance of sori and the recruitment on clean artificial substrate beneath adult specimens. The pattern of recruitment was linked to the retreat of visible sori (i.e. spore release) and a seasonal component unrelated to the fertility of the adults. The formation and the retreat of visible sori are processes that seem synchronized along the south coast of Norway, and the link between sori development and recruitment may therefore suggest that the potential for *S. latissima* dispersal is relatively large. These results support the notion that the production and dispersal of viable spores is unlikely to be the bottleneck preventing recolonization in the south of Norway, but studies over larger temporal and spatial scales are still needed to confirm this hypothesis.

## Introduction

Kelps, seaweeds, and seagrasses provide important ecosystem services in coastal areas, and the loss of these macrophytes is a global concern [1]–[5]. In Norway, the disappearance of the perennial kelp *Saccharina latissima* has raised considerable concerns both within and outside the scientific community [6]. The Norwegian authorities have called on researchers for plans of action, and some of the preliminary suggestions involve measures aimed at facilitating natural recolonization. Successful kelp recruitment is a prerequisite for recolonization, yet there are few studies of *S. latissima* recruitment *in situ*.

Kelps exhibit life histories in which large diploid sporophytes (benthic spore producing stages) alternate with microscopic haploid gametophytes (benthic sexual stages) via flagellated spores (planktonic dispersal stages). The production of spores in *S. latissima* is significant, and kelp spores may disperse over great distances [7], [8]. Although most settle near the mother plants [9], [10], large-scale oceanographic processes may serve as key drivers of connectivity (exchange of genetic material) between kelp populations [11]. Kelp recolonization of barren grounds [12] and colonization patterns on artificial reefs [pers.com. Hartvig Christie] in Norway are also consistent with long-distance dispersal of *S. latissima*, and rapid forest recovery after *S. latissima* deforestation in the past indicate that natural recolonization was once possible [6]. Today, turf algae communities loaded with sediments seem persistent in most of the deforested areas, while *S. latissima* remains absent.

*Saccharina latissima* growing along the Skagerrak coast of Norway initiate the formation of sori (fertile tissue) in October, and sori are usually observed until spring (March–April) [13]. The distinct pattern of sori development in Norwegian *S. latissima* (similar to that of *Pterygophora californica* in the study by Reed et al. [14]) may indicate reproductive synchrony among adjacent populations. Supporting this notion, previous observations indicate that the amount of *S. latissima* recruits *in situ* varies considerably through the kelps' reproductive period [Henning Steen, unpublished]. This result may be important to validate, because a high degree of reproductive synchrony has a positive effect on the dispersal potential of kelp populations [10], [14].

The present study recorded *S. latissima* recruitment on clean artificial substrate directly beneath adult sporophytes (parent kelps) throughout a reproductive period. The aim of the study was to determine the extent of coupling between the development of fertile tissue (sori) on the parent kelp and recruitment, and to investigate whether the recruitment showed seasonal variation that was unrelated to the fertility of the parent sporophytes. The influence of such a seasonal component would mean that the probability of transition from spore to sporophyte varied throughout the reproductive period. To aid the interpretations, the recruitment on substrate that had been exposed to *in situ* spore settlement for one, two and three months was recorded. In addition, a series of laboratory experiments was performed to investigate recruitment both early and late in the reproductive period of the kelp. The aim was to investigate the seasonal differences in the time-related pattern of recruitment following spore release, because such differences would affect the proportion of recruits settling directly beneath the kelp *in situ*.

## Materials and Methods

### Ethics statement

The deployment of rigs, the sampling and the laboratory work was executed in accordance with Norwegian regulations. The study did not involve endangered or protected species and the research complied with all applicable Norwegian laws.

### Field study

The pattern of recruitment and the extent of coupling to sori development on the parent kelp was investigated in a field experiment.

Fifteen adult (>1 yr) *Saccharina latissima* individuals were sampled from a persisting population just outside Grimstad on the south coast of Norway (58°19′N, 8°35′E, WGS84 datum). The kelp plants were mounted on separate rigs and deployed at three sites inside a deforested area (>30 m between rigs and >300 m between sites). The specimens were kept at a 3 m depth and monitored for approx. one year to detect seasonal patterns of sori formation. Sori formation was estimated as the percentage cover of the kelps' blade area. A narrowing of the blade somewhere close to the distal part was visible on all blades. This narrowing represents the transition between two growth periods. The blade area from the base of the blade to this narrowing was considered new tissue (<1 year old), while the remaining distal part of the blade was considered old tissue (probably >1 year old).

Trays with a series of wrenched holes were mounted on the rigs at a 5 m depth (2 m below the kelp), one tray on each rig. Removable plexiglass quadrats (3×3 cm, termed tiles hereafter) were attached to the trays with polypropylene screws. The surface of the tiles had been ground with sandpaper to make them suitable for *S. latissima* spore attachment. After one, two, and three months' deployment in the field, the tiles were collected. The comparison of recruitment on tiles that had been in the field for different durations allowed for an evaluation of density effects. The collection of tiles, and the subsequent deployment of new ones, was executed every month from December 2007 until May 2008. After collection, the tiles were stacked onto wrenched bolts (approx. length of 10 cm) with slender spacers in-between each tile (1–2 mm spacing). Clean tiles were placed at each end of the stacks to protect the spores/recruits on the first and last recruitment tile in each stack. Each stack was kept tight on the bolt with a nut to ensure minimal disturbance of the tile surfaces during transport to the laboratory. The samples were protected from direct sunlight at all times and transported in dark coolers containing seawater. In the laboratory, the tiles were separated and put in individual petri dishes containing IMR/2 medium. The petri dishes were kept at a temperature of 12°C (close to the optimal temperature for gametophyte and sporophyte growth [2]) in a 16 : 8 hour light-dark cycle under 50 µmol photons *m* ^−2^ *s* ^−1^ for approximately one week. *S. latissima* recruits (juvenile sporophytes) on each tile were finally counted using a light microscope.

The parent kelps were trimmed down to 1 m length at each collection event. The trimming served two purposes: 1) the blade area of each kelp was kept similar in size throughout the experiment, making comparisons of sori covers easier and 2) the disturbance caused by long blades sweeping the spore collecting devices was avoided. Trimming was, however, seldom needed in the reproductive period as this is also a period of minuscule growth in *S. latissima*.

### Laboratory study

In order to interpret the seasonal aspect of the field experiment, a laboratory experiment was conducted. The aim was to investigate the time-related pattern of recruitment in the minutes and hours following spore release, and to detect whether the pattern changed during the reproductive period of the kelp. Such a change would affect the recruitment observed directly beneath the parent kelp, and thus be vital for the interpretation of the field results.

Mature sporophytes (6 individuals) with visible sori were collected from the persistent population just outside Grimstad on the south coast of Norway in January 2009, April 2009, February 2011, and March 2011. A spore solution (mixture of kelp spores and seawater) was prepared from the fertile tissue, following the same procedure at every sampling event. From each kelp individual, one sample of fertile tissue (6 *cm* ^2^) was taken. The samples were rinsed in fresh water for five seconds and blotted dry with paper towels for two minutes before they were submersed in 1 L of filtered seawater. The tissue samples were left in the water for 45 minutes (to ensure spore release), and spores from the different tissue samples were mixed. To create the spore solution, 110 mL of the spore mixture was added to 990 mL of filtered seawater. This dilution was performed because induced spore release tends to cause very high concentrations of spores, and very high concentrations are more difficult to work with (based on own experience with kelp cultivation).

The number of spores was counted manually in six subsamples of the solution using a hemocytometer, and the spore concentration was estimated. A 25 mL sample of the spore solution was added to four petri dishes containing one standard cover slip for microscopy each (18 mm×18 mm). Each petri dish represented the onset of one series. At given time intervals, the spore solution in each petri dish was carefully poured into a new petri dish with a new cover slip. The transfer was executed at 10 min intervals for one hour, at 20 min intervals for the second hour, at 30 min intervals for the next four hours, every hour for four subsequent hours, every second hour for six hours, and, finally, every third hour until the series was discontinued after 25 hours. The time schedule was based on experiences with a pilot study in January 2009. After every spore solution transfer, the preceding petri dish was filled with growth medium (IMR/2) to ensure growth of the settled spores. All cover slips were kept at 6°C in a 16 : 8 light-dark cycle under 50 µmol photons *m* ^−2^ *s* ^−1^ (PAR). The average sea water temperature at a 10 m depth was 5.04 (±1.2 SD) from January to April, so the temperature in the culture room was similar to natural conditions.

After one month in culture, the number of recruits (juvenile sporophytes) was counted using a light microscope (10×10 magnification), scrolling one lane across each cover slip. No particular edge effect was observed. If present, but undetected, such an effect would be likely to affect all samples and be of little consequence for the overall results. All recruits within a lane were therefore counted. The area on each cover slip in which the recruits were counted (1.25 mm×18 mm) will hereafter be called the counting field. The rate of recruitment was determined by dividing the number of recruits observed in a counting field with the amount of time spore settlement had been allowed to occur on that particular slip (the interval duration).

### Method development for the laboratory study

Two different treatments were tested in the pilot project in January 2009. Spore solutions were first poured into petri dishes containing two cover slips. One of the cover slips in each dish was then gently removed and placed in a new petri dish containing growth medium. The remaining cover slip in the old dish was treated as described in the previous paragraph, and the spore solution was transferred to a new petri dish containing two additional cover slips. The differences in recruitment between the two treatments were analyzed using a generalized linear model (GLM) of the Poisson family. The analysis proved the effect of treatment to be nonsignificant (P>0.1).

### Statistical analyses

All statistical analyses were conducted with the R statistical software [15]. The recruitment in the field was analyzed with hurdle models based on the *pscl* package [16]. The recruitment patterns observed in the laboratory were analyzed with generalized linear mixed models (GLMM) via PQL, based on the *MASS* package [17], while the spore concentrations were compared using a GLM of the Poisson family. Datasets and R-scripts are publicly available in a GitHub-repository (http://bit.ly/SAndersen2013).

The numbers of recruits observed on the field tiles were analyzed in relation to the time of tile deployment (November–May), the duration of deployment (1, 2, or 3 months) and sori cover on the parent kelp. The traditional methods for analyzing count data (such as the GLM of the Poisson or Quasipoisson family) was considered, but the data contained many zeros, and the model predictions were over-dispersed. The use of a hurdle model was a much better option for several reasons. The idea underlying the hurdle model is that the observed response (in this case, the number of kelp recruits) is the result of two ecological processes [18]. In the context of the present study, one process was assumed to cause the presence or absence of recruits (i.e., the presence or lack of spores in the seawater), and where recruits were present, a second process was expected to influence the actual number of them (e.g., spore viability, grazing, or density controlling mechanisms). Model selection was performed using the Akaike Information Criterion as a measure of relative goodness of fit [19]. The model residuals were plotted and inspected, especially in relation to station and rig, to detect any patterns suggesting that mixed modeling was required. No residual pattern was shown in relation to either factor, and mixed effect modeling was therefore deemed unnecessary. Hence, the data from the three different stations were pooled.

The rate of recruitment observed in the laboratory experiment was analyzed in relation to time after spore release (Time), the number of recruits left in the spore solution above the counting field (Density), and the timing within the reproductive period of *S. latissima* (early or late). The main goal was to look at the seasonal differences, and year (2009 or 2011) was therefore included as a random factor. The density of spores could not be measured in the solutions during the experiment. Instead, a proxy was calculated by adding the number of recruits observed in a counting field to the number of recruits observed in the subsequent counting fields within the series. Hence, “Density” in the models reflected the number of potential recruits left in the solution at the beginning of the given interval. How interval duration affected the rates was not known, but stochastic processes changing with duration could potentially influence the results. To look at the effects of season, time, and the density proxy, the data were grouped according to interval duration and one analysis was performed per data group. The rates appeared to be gamma distributed, and GLMMs of the Gamma family was therefore used. Finally, the residuals were inspected to detect any autocorrelation within the series, but no patterns were found, and the analyses were deemed appropriate.

## Results

### Field study

**Figure 1:**
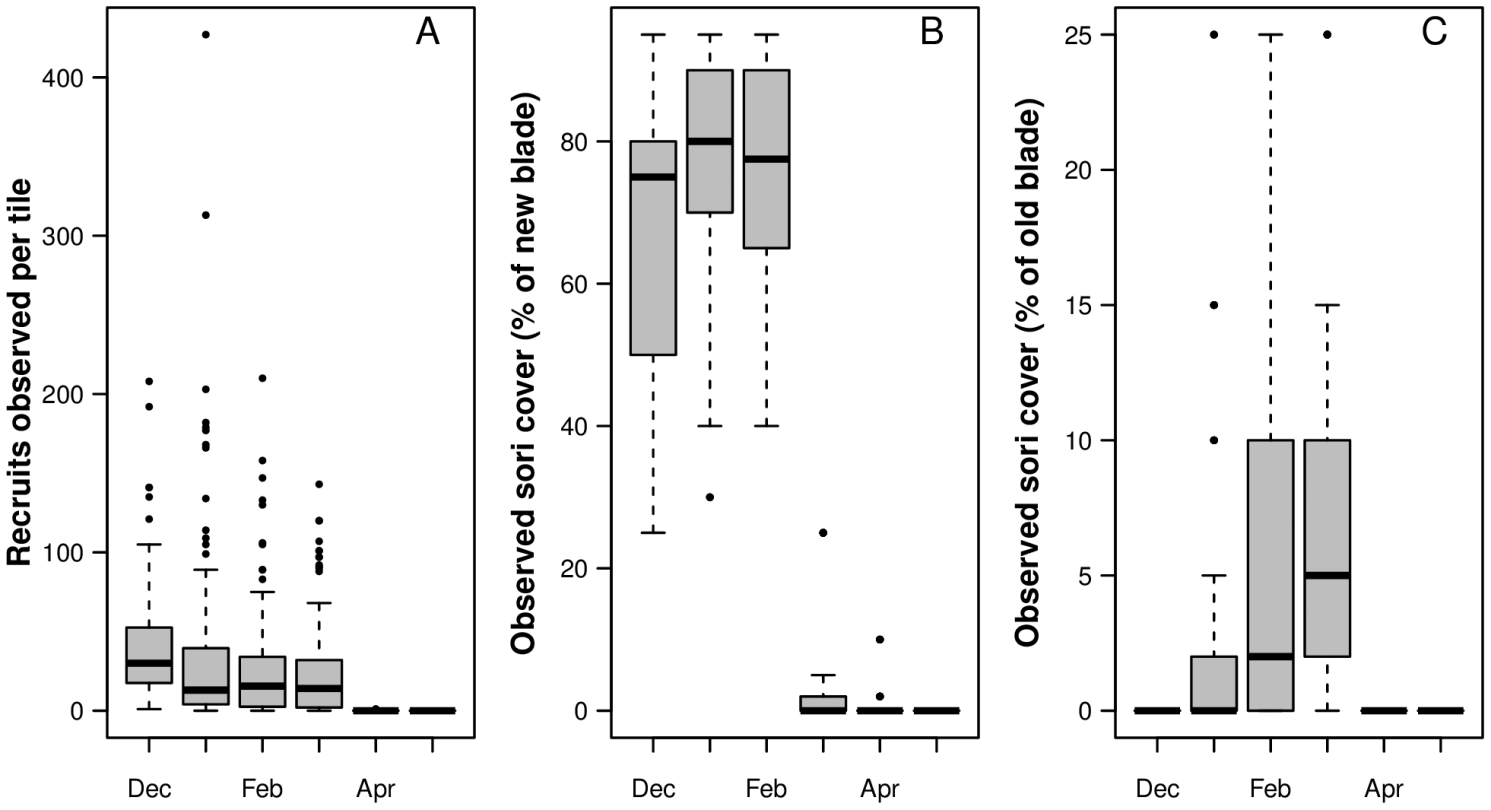
Summary of results from the field experiment (December 2007–May 2008). Boxplot A shows the number of recruits observed on the tiles. Boxplot B shows sori coverage observed on the new parts of each blade, while C shows sori coverage observed on the older parts of each blade.

Scattered spots of visible sori were observed on all kelp sporophytes in November, when the field study was initiated. By December, the spots had grown to larger areas, and the development of visible spore forming tissue continued until February. New parts of the blade formed sori early, while older (more distal) parts of the blade formed sori late in the reproductive period (Figure 1 B and C). In April, all of the dark areas were gone, indicating that most of the sporangia (the compartments containing spores) were empty.

Recruitment was observed throughout the period when the kelps had visible sori (Figure 1 A), suggesting that spores were released from the adult plant continuously. The hurdle model, however, revealed that the recruitment on the tiles varied (Table 1).

**Table 1:** Hurdle model selection.

*Model*	*HP1*		*HNb1*		*HNb3a*	
Which	Count	Zero	Count	Zero	Count	Zero
Family	Pois	Bin	Negbin	Bin	Negbin	Bin
Duration (D)	**0.002**	0.154	0.251	0.154	0.233	0.154
Month (M)	**≪0.001**	**≪0.001**	**<0.001**	**≪0.001**	**<0.001**	**≪0.001**
Sori new (SN)	0.0705	**≪0.001**	0.218	**≪0.001**	0.109	**≪0.001**
Sori old (SO)	**≪0.001**	**0.012**	0.065	**0.012**	**0.010**	**0.012**
D x M	**≪0.001**	0.058	0.119	0.058	0.132	0.058
SN x SO	**≪0.001**	**0.016**	0.613	**0.016**		**0.016**
AIC	20833		5372		5370	

Models with different error distributions were tested (Pois = Poisson, Bin = Binomial, Negbin = Negative Binomial). The Zero part of each model predicts the probability of recruitment, while the Count part predicts the number of recruits given Zero≠0. Significant P-values are indicated by bold formatting. The interactions between Month and Sori (both new and old) were not significant in any of the models. Because it had the lowest AIC value, the best model was *HNb3a*.

The hurdle model is based on the assumption that two processes determine the response. In this study, one process was assumed to determine the probability of finding recruits, and *if* recruits were found, another process was assumed to have determined the actual number of recruits. The duration of tile deployment (1, 2, or 3 months in the field) did not seem to significantly affect either the probability of observing kelp recruits on the tiles nor the count (Table 1). In contrast, the time of deployment greatly affected both the probability of recruitment and the number of juvenile kelp plants that was observed on a tile. The amount of new blade sori affected the probability but not the number, while the amount of old blade sori affected both. The model accounted for approximately 68% of the observed variation.

**Figure 2:**
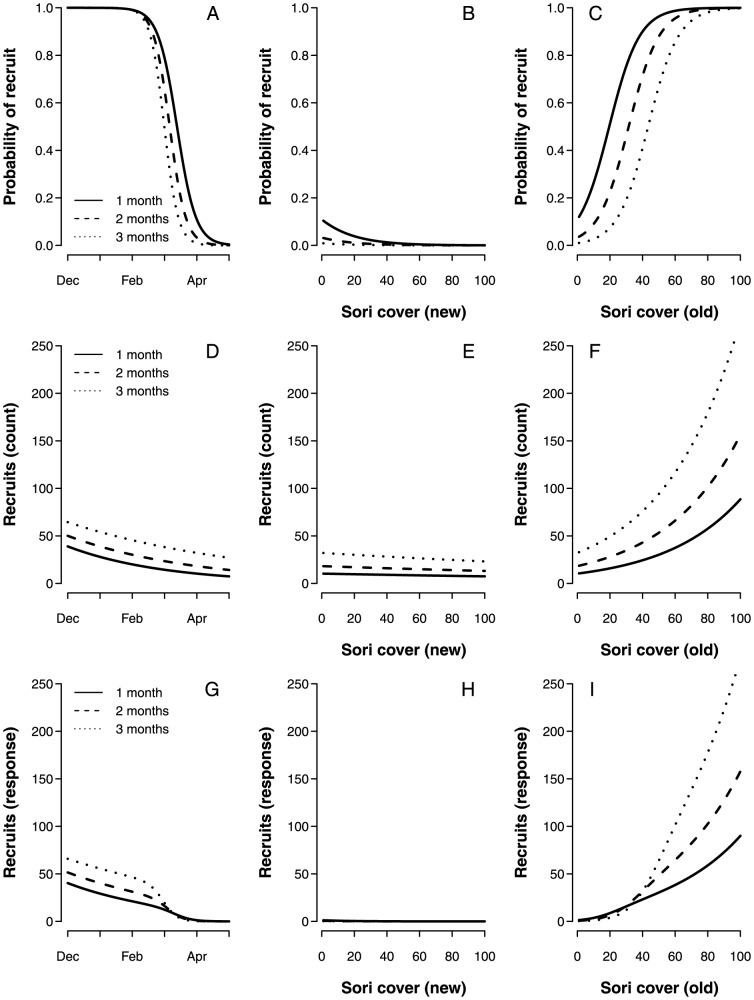
Graphical presentation of the model predictions in relation to varying time of tile deployment, sori cover on new tissue, and sori cover on old tissue. Different line types represent different durations of tile deployment. A, B, and C show the predicted probability of observing recruitment. D, E, and F show the predicted number of recruits where recruits are present, while G, H, and I show overall count predictions.

To visualize the effects of each continuous explanatory variable in the model, a set of predictions were made based on a constructed dataset in which only one continuous variable was allowed to vary at a time. The outcome of these predictions are shown in (Figure 2).

Both the presence and density of recruits were related to time of deployment and worked in favor of higher recruitment densities early in the reproductive period (in the Northern Hemisphere winter) (Figure 2 A, D, and G). The fertility of the parent kelp also seemed important, but the relationship depended on which parts of the blade were covered by sori. The predicted effect of increased cover on the new part of the blade (<1 yr) was negative in relation to both the probability of observing recruits and the number of recruits (Figure 2 B, E, and H), though not statistically significant in the latter case (Table 1). In contrast, the predicted effect of increased sori cover on the old part of the blade (>1 yr) was strongly positive (Figure 2 C, F and I). The extent of this effect did, however, depend on the amount of new blade sori (see interaction term in Table 1).

In summary, the recruitment of *Saccharina latissima* was highest early in the reproductive period. Recruitment was low beneath kelp plants that still contained spores in the newest part of the blade (i.e., had visible new blade sori), while recruitment was high beneath kelp plants that had begun to develop spores in the older part of the blade.

### Laboratory study

The concentration of spores in the spore solutions was 1 349 000 mL^−1^ early in the kelps' reproductive period and 17 240 mL^−1^ late in the reproductive period in 2011. The volume of spore solution directly above each counting field at the initiation of the experiment was 450 mm^3^, which translates to 0.45 mL and brings the estimated amount of spores potentially settling in one whole series up to numbers of 607 050 and 7 523 in the respective periods. It was assumed that half of the spores were female and that all females produced sporophytes (although both sex-ratios and success rates may vary somewhat [20], [21]), and the number of potential recruits was estimated to be 303 525 (±20% SD) and 3 761 (±14% SD), respectively. The number of realized recruits observed in the series was far less than the estimated potential both months, approx 0.6% of the expected number early in the reproductive period and 64% later on. Despite the lower spore concentration in the spore solution, the recruitment of juvenile sporophytes was therefore much higher late in the reproductive period (2 399±8% SD as compared to 1 739±12% SD earlier on). More realized recruits were observed in 2009, but the concentrations of spores in these solutions were unfortunately not measured.

The rates of recruitment were similar in both seasons, except for after 6 hours, when significantly higher rates were observed late compared to early in the reproductive period (in the Northern Hemisphere winter). Seasonal differences were also observed in the relationships between recruitment, time, and density in some of the duration intervals (Table 2, see also Figure S1).

**Table 2:** GLMM models of recruitment in the lab.

Hours after initiation	<1 hrs	1–6 hrs		>6 hrs	
Interval duration	10 min	20 min	30 min	60 min	120 min
Intercept (late)	0.536	0.728	**0.041**	**<0.001**	0.379
Season (early vs late)	0.154	0.539	0.053	**<0.001**	0.319
Time (late)	**<0.001**	0.333	0.437	**0.001**	0.970
Density (late)	**0.008**	0.816	0.465	**0.002**	0.791
Time x Season (early vs late)	0.0506	0.886	0.411	**0.001**	0.792
Density x Season (early vs late)	0.8051	**0.009**	**<0.001**	0.065	0.407
Time x Density	**<0.001**	0.208	0.871	**0.027**	0.053

One model was made per interval duration. The interval durations were correlated with Time because the intervals were shorter early in the experiment and subsequently longer as time progressed. After the 120 min intervals were completed (16 hours into the experiment), too little settlement was observed to perform any analysis. Season refers to early (Northern Hemisphere winter) and late (Northern Hemisphere spring) in the reproductive period. Year was included as a random factor. Significant P-values are indicated by bold formatting. Plots showing the effects of the significant parameters on the predictions are presented in the Supporting Information.

**Figure 3:**
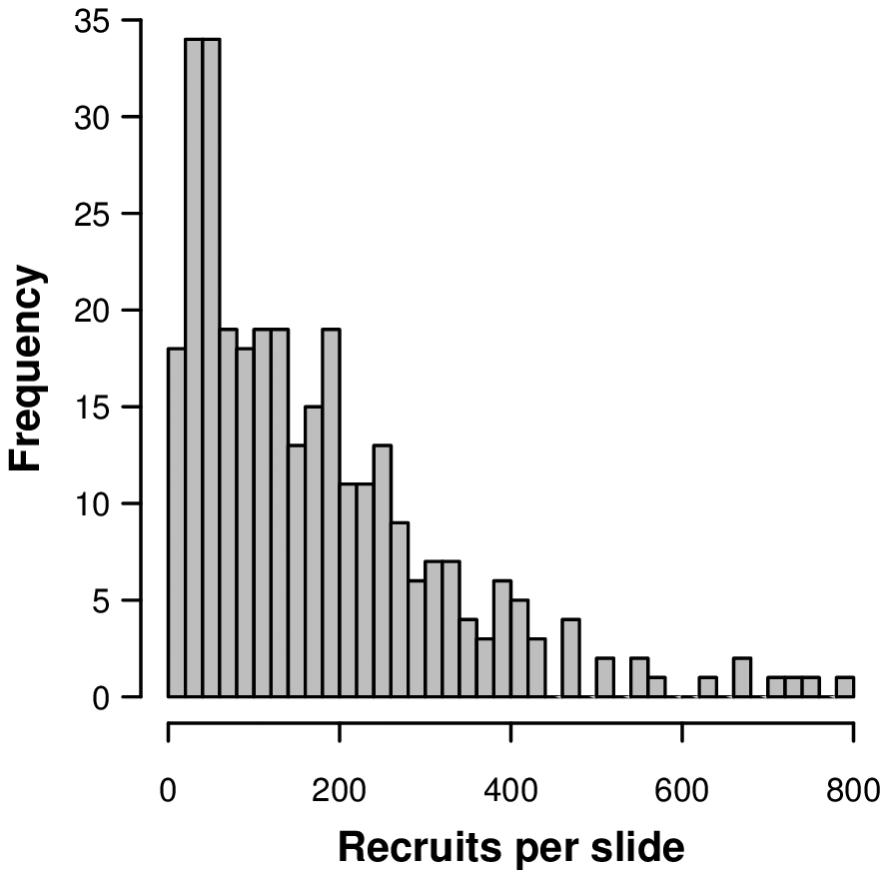
Histogram showing the frequency of kelp recruit counts on the cover slides.

The rate of recruitment on the cover slips was generally high shortly after the spores had been released from the parent kelp and subsided very rapidly. The rate then stabilized and remained almost constant within the 20 minute and 30 minute interval groups, i.e., from 1–6 hours into the experiment. This pattern was similar both seasons. After 6 hours, the rate subsided with time late in the reproductive period, while it remained almost constant early in the reproductive period of the kelp (except for at a very high density of potential recruits) (Table 2, see also Figure S1). These results indicate that seasonal differences in the time related recruitment patterns did occur and that the effect appeared quite a long time after the spores had been released. After 10 hours, the recruitment became sporadic, and no significant differences were found between the cover slips. Because the amounts of unsuccessful spores were quite large in both seasons, this indicates that the probability of transition from spore to recruit may have been reduced after 10 hours in the laboratory. The same pattern could have been the consequence of spore depletion and density limits on recruits early on. However, the frequency of recruit counts throughout the study was highest at the low end of the scale (Figure 3), so in most cases, the cover slides were probably large enough for the accumulated spores to develop.

The density of potential recruits in the spore solution affected recruitment rates, but had a significant effect on the time-related pattern (Time x Density in Table 2) only at the initiation of the experiment. As time progressed within the first hour, the predicted effect of Density became increasingly positive (Figure S2). This result may indicate that density-regulating mechanisms (either in mortality or settlement behavior) occurred shortly after spore release during both seasons, but caution is recommended in the interpretation of these results. There are probably better and more ecologically relevant ways to assess density-controlling mechanisms in the recruitment of *S. latissima*. In the time span from 1 to 6 hours after the initiated spore release, high densities of potential recruits had a positive effect on recruitment, but this effect was significant only early in the reproductive period. The rates of recruitment thus seemed rather stable within this time frame (that is, within the 20 and 30 minute intervals).

In summary, lower spore densities but more recruits were found late compared to early in the reproductive period of *S. latissima*. The time-related patterns of recruitment were however similar in both periods (except for after 6 hours).

## Discussion

*Saccharina latissima* sporophytes translocated into a deforested area in Skagerrak were able to produce fertile tissue and release viable spores. Dense *in situ* recruitment of juvenile *S. latissima* was observed on clean substrate, which further indicated that environmental conditions in the water column did not prevent kelp recruitment. The recruitment of juvenile sporophytes *in situ* can be viewed as the result of two processes: one process that determines the presence of recruits and another process that determines the number of recruits. The present study shows that both processes depend, to different degrees, on the development of spore-producing tissue (sori). Furthermore, the study indicates that a seasonal component independent of spore production in the parental plants also affects recruitment.

The recruitment of juvenile *S. latissima* sporophytes in the field was highest in December and January. Seemingly contradictory, the laboratory study showed much lower kelp recruitment during the peak season than later in the reproductive period of the kelp. This result was not only contradictory but also counter-intuitive as a much higher density of spores was found in the spore solution prepared at that time. Spores must, however, be contained in the sori for a certain amount of time to fully develop [22]. A spore contained in any sporangium was therefore more likely to be fully developed at the end of the reproductive period (March/April) than earlier on (January/February). Because forced release by freshwater rinsing and drying is an effective way of inducing spore release [23], it was assumed that most of the sporangia within the sori were emptied in the process. And the greater number of fully developed spores contained in the sori collected, may thus have accounted for the greater recruitment success in the laboratory late in the reproductive period of the kelp.

The first appearance of sori on the parental *S. latissima* tended to be located in the middle of the frond, which was considered new tissue. The probability of observing recruitment in the field increased as the sori cover on the new parts of the parental plant disappeared. When sori disappear, it is because spores are released, so increased recruitment was not a surprising effect. Older tissue developed sori later than new tissue (Figure 1 C *vs* B), and increased sori cover on the old parts of the parental plant seemed to increase both the probability of recruitment and the number of recruits beneath the plant. However, these effects may not be linked to old blade sori cover *per se*. As sori develop in old tissue, increasing amounts of spores contained in the younger parts of the blade would have matured and therefore been released at increasing rates. The significant interaction (SO x SN in the hurdle model) also indicated that the correlation between recruitment and visible sori in the old parts of the kelp plant depended on the visibility of sori in the newer tissue. The high recruitment coinciding with sori development in the parents' old tissue was therefore largely a consequence of spore release from the younger tissue.

The probability of finding recruits in the field dropped markedly between March and April, independently of sori cover (see Figure 2 A). This result indicated that some seasonal mechanism, unrelated to the fertility of the parental sporophyte, affected the recruitment on the tiles. The explanation could have been seasonal differences in the physiology of the released spores, but the results from the laboratory study indicate that the differences were small. The time-related recruitment patterns were almost identical in both seasons during the first 6 hours of the laboratory experiment. Because the recruitment tiles were located directly beneath the parent kelp, this was also the time frame in which differences would have been most likely to affect the results in the field. The seasonal component may be explained by variation in the interactions between physical and biological factors and the kelps microscopic stages in the field [24], but this discussion lies well beyond the scope of the present study.

The duration of tile deployment in the field was expected to increase the number of kelp recruits because the tiles would have had more time to accumulate spores. The effect was, however, relatively small and statistically insignificant throughout the study, which indicates that some sort of density-controlling mechanism was constantly at play. This mechanism could be related to the experimental design: Because the tiles were always clean when deployed, but probably accumulated sediment, bacteria, and other organisms in the field, the quality of the tile as substrate may have been progressively reduced during deployment. Other possible explanations are intra- and inter-specific competition, but this discussion also goes beyond the scope of the present study.

The extent of connectivity along the south coast of Norway is not known, but previous records [6] indicate that dispersal from remnant populations and subsequent recovery was once possible. Connectivity between kelp populations is reinforced by reproductive synchrony because higher densities of spores in the currents increase the probability of long-distance dispersal [14]. The seasonal development and demise of visible sori in *S. latissima* are processes that largely overlap along the south coast of Norway [13], [25, pers. com. Stein Fredriksen]. The present study showed that *S. latissima* recruitment was tightly linked to this pattern and may therefore also support the notion that the potential for connectivity between *S. latissima* populations in Norway is high. If this is true, forest regeneration through facilitation of natural recolonization may be feasible because remnant populations still exist and the environmental conditions in the water seem to permit it.

Whether the present-day bottom conditions permit kelp recruitment in the deforested areas is, however, a different matter. Sediment covers and turf algae communities have been shown to impair kelp recruitment in other areas of the world [26]–[28]. The persistence of sediment-loaded carpets of turf algae and the lack of forest recovery in most deforested areas in Skagerrak [6] suggest that this may be the case in Norway as well. Other mechanisms may also obstruct the transition from juvenile to mature kelp: The juvenile sporophytes may, for instance, experience high temperature stress and overgrowth by epiphytic organisms from June to September (the Northern Hemisphere summer). These stressors may reduce the kelps' chance of survival [13], [29] and thereby hinder the formation of populations able to sustain themselves. To evaluate management strategies involving facilitation of kelp recruitment and the probability of their success, further studies on the impacts of multiple stressors are needed.

## Supporting Information

Figure S1 **The significant effects on the GLMM model predictions in the 20, 30 and 60 minute interval groups.** Solid lines represent effects late, while dashed lines represent effects in early in the reproductive period of *S. latissima*. Predicted recruitment increased with increasing density of potential recruits, and the effect was significantly different early compared to late in the reproductive period in all three interval groups. Predicted recruitment decreased with time in the 60 minute interval group, except for during the reproductive peak season at high densities, when predictions increased with time.
(TIFF)

Figure S2 **The effect of time on the GLMM model predictions varied with the density of potential recruits in the 10 minutes interval group.** The positive effect of time at the highest densities may suggest the possibility that recruitment was limited by substrate availability at the initiation of the experiment.
(TIFF)
